# The Landscape of Videofluoroscopy in the UK: A Web-Based Survey

**DOI:** 10.1007/s00455-020-10130-1

**Published:** 2020-05-16

**Authors:** J. K. Benfield, E. Michou, L. F. Everton, C. Mills, S. Hamdy, P. M. Bath, T. J. England

**Affiliations:** 1 Division of Medical Science and Graduate Entry Medicine, School of Medicine, University of Nottingham, Nottingham, UK; 2 Centre for Gastrointestinal Sciences, The University of Manchester, Manchester, UK; 3 Division of Clinical Neuroscience, School of Medicine, University of Nottingham, Nottingham, UK; 4 Academic Unit of Health Economics, University of Leeds, Leeds, UK; 5 Division of Diabetes, Endocrinology and Gastroenterology, School of Medical Sciences, Faculty of Biology, Medicine and Health, University of Manchester, Manchester, UK; 6 Stroke Trials Unit, Division of Clinical Neuroscience, School of Medicine, University of Nottingham, Nottingham, UK; 7 Speech Language Therapy, TEI Western Greece, Patras, Greece

**Keywords:** Dysphagia, Videofluoroscopy, Swallowing, Deglutition, Deglutition disorders

## Abstract

Videofluoroscopy (VFS) is considered one of the gold-standard assessments of swallowing. Whilst guidelines for the application and conduct of VFS exist, their translation into clinical practice remain challenging. To build a greater understanding on how VFS clinics operate in the UK. A web-based survey was shared with speech and language therapists (SLTs) working in VFS clinics via professional networks and social media from October 2018 to January 2019. 101 responses were received. Two thirds of clinics were SLT-led, with the majority of clinics being run by two SLTs (73.6%) and a radiographer (95.5%) also known as radiologic technologists, diagnostic radiographers and medical radiation technologists. Less than 50% of radiographers had received specialist training. Around half of the clinics used a standard assessment or analysis protocol and 88.1% a rating scale. Set recipes for a range of textures were used in 53.4% of VFS clinics. Barium and water soluble contrasts were used, but only 15.8% knew the concentration of contrast used. The most commonly reported VFS pulse and frame rate was 15 per second. There was evidence of a lack of SLT knowledge regarding technical operation of VFS. Screening times varied from 0.7–10 min (median 3 min, IQR 2.5–3.5). Around 50% of respondents reported quality issues affecting analysis. In a survey of UK SLTs, translation of VFS guidance into practice was found to be limited which may impact on the quality of assessment and analysis. Collaboration with radiology, strengthening of guidelines and greater uptake of specialist training is deemed essential.

**Electronic supplementary material:**

The online version of this article (10.1007/s00455-020-10130-1) contains supplementary material, which is available to authorized users.

## Introduction

Videofluoroscopy (VFS) is an instrumental tool for assessing the physiology and safety of oropharyngeal swallowing as well as oesophageal clearance. During the assessment, a patient is asked to swallow different volumes and consistencies of foods and drinks mixed with a radio-opaque contrast to allow visualisation on X-ray. Swallowing strategies and manoeuvres are also trialled to compensate for physiological impairments in the swallowing. It is one of the ‘gold standard’ assessment tools in the field because both anatomical hallmarks and swallowing physiology can be visualised in real-time. VFS is usually carried out by a speech and language therapist (SLT) and a radiologist and/or a radiographer. Radiographers, also known as radiologic technologists, diagnostic radiographers and medical radiation technologists are healthcare professionals who specialise in the imaging of the human anatomy for the diagnosis and treatment of pathology.

In 2006, a UK survey conducted by Power and colleagues revealed variability in the conduct of VFS clinics with a range of assessment materials and protocols being used, an under use of research evidence in the preparation of assessment materials, the lack of specialist training for SLTs and radiographers and limited interdisciplinary working [1]. Since then the Royal College of Speech and Language Therapists (RCSLT) updated their position paper [2] giving broad guidance with regards to the technical set-up, assessment and analysis methods and working with the radiologist or radiographer to optimise image quality whilst keeping radiation exposure as low as reasonably achievable (ALARA).

Further research has also been conducted into VFS processes that can have an impact on the interpretation of findings. Several studies have compared different temporal resolutions of VFS and the effect on interpretation of physiological and aspiration events [3–5]. A number of studies have documented the importance of using the correct concentration of contrast and consistency of materials to ensure reliable VFS interpretation [6–8].

Little is known about how UK practice has changed since the 2006 survey and whether RCSLT VFS guidelines or recent research has filtered into clinical practice. The aims of this study were to build an up-to-date understanding of how UK VFS clinics are conducted, how guidelines and research have been embedded into practice and how this compares to the findings of 2006.

## Methods

### Procedure

A web-based self-administered survey was devised by expert SLTs using Google Forms. It was piloted with three other expert VFS clinicians and their feedback was used to improve the question content and layout. Questions covered four main sub-topics: clinic governance and staffing, VFS equipment set-up, assessment methods and analysis methods (Supplementary Online Material 1).

### Participants

The survey was shared via professional networks and social media between October 2018 and January 2019. SLTs who were involved in VFS clinics were asked to complete the survey for their clinic. This was on a voluntary basis; respondents did not receive any compensation for their participation. Dissemination of surveys in this way allows wide coverage of a population. However, using this method means that the size of the population that receives it cannot be determined.

### Ethics

University of Nottingham Faculty of Medicine Research Ethics Committee assessed that a full review by the committee was not indicated due to the nature of the work being a national service evaluation (University of Nottingham Ethics Reference No: 136-1810). Participants provided informed consent prior to completing the survey.

### Statistical analysis

Data were analysed using SPSS 24 and Microsoft Excel 2016, descriptive statistics were used to describe and summarise the data. The Chi-square test was used to test associations between categorical variables. Several questions allowed a free text or ‘other’ response with opportunity for free text, which resulted in a spectrum of responses to the same question. For example, screening times were given in whole values, ranges, minimum and maximum values. Responses were grouped into categories where appropriate for the purposes of analysis. However, for the purposes of analysis of the banding (grade) of SLTs, minimum band SLT was taken and if a range was given, the minimum value was assigned.

## Results

One hundred and four responses were received from SLTs. Two responses were excluded from the main analysis as these were community SLTs, not involved with running of the VFS clinics but referring patients to local services. One response was excluded because it related to a clinic that used VFS to analyse speech production. One response was received from The Channel Islands, which is outside of the United Kingdom, but due to close links to the mainland hospital trust, the response was included.

On five occasions more than one response was received from the same hospital and it was unclear whether they referred to the same or a different clinic within the hospital due to variations in the responses. The responses were included with the assumption that they related to a second clinic within the hospital.

In total 101 responses were analysed. Nine respondents gave details of a second clinic with a different staffing configuration; therefore, for data related to staffing, the total responses analysed were 110.

### Region

**Fig. 1:**
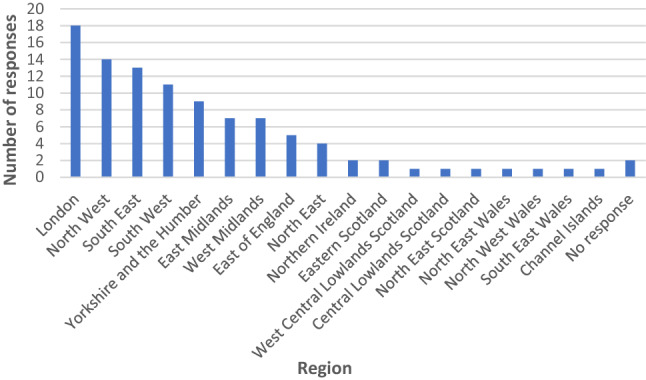
Responses from regions around the United Kingdom and the Channel Islands

Responses were received from across the United Kingdom, although more responses were received from England. Figure 1 shows the number of responses per region.

### Sample

The UK has Government-funded medical and health care services that everyone living in the UK can use without being asked to pay the full cost of the service. Components of this National Health Service (NHS) are organised into clinical care groups (CCGs) overseeing primary care (including general practice) and secondary care trusts providing acute and specialist services. Responses were received from 73 acute NHS trusts, representing 54.8% of the 135 acute hospital trusts in the UK. However, there are likely to be several VFS clinics across each trust. The response rate and response bias were not able to be accurately estimated because it is unknown how many SLTs involved in VFS clinics received notification of the survey via social media adding to the fact that the number of VFS clinics nationwide is not clearly defined.

### Clinical Governance and Staffing

The number of patient appointments ranged from 0 to 12 per week across services with a mean of 4.1 (SD 2.8) slots. A small percentage of responses (4%) related to paediatric clinics. As numbers were small, associations with other responses such as staffing, technical set-up or assessment and analysis could not be derived.

#### Speech and Language Therapists

Two SLTs were reported to be present in 73.6% (81/110) of VFS clinics, one SLT in 23.6% (26/110) of clinics and in 1.8% (2/110) of clinics three SLTs are present. The median minimum band of SLTs in clinic is band 6 (range 5–8). The majority of clinics operate with a minimum band 6 or 7. When there is only one SLT in clinic, 73.1% (19/26) are band 7 or above. The UK uses a pay banding system to grade the levels of responsibilities of SLTs (range band 5–8). A band 6 SLT will usually have at least 2 years’ experience and a band 7 at least 5 year’s. There was a significant association between region and number of SLTs running clinics [*Χ*^2^(90) = 141.8, *p* < 0.001]. East Midlands have a significantly higher percentage of clinics run with only 1 SLT compared to the rest of the UK.

#### Radiologists

A radiologist is always present in 28.3% (31/110) of clinics, sometimes present in 4.5% (5/110) of clinics and not present in 66.3% (73/110) of clinics. If not or only sometimes present, a radiologist is available to review images 85.9% (67/78) of the time. A radiologist is significantly more likely to be present in clinics with only one SLT than with two SLTs [*Χ*^2^(16) = 44.7, *p* < 0.001].

#### Radiographers

Radiographers are present in 95.5% (105/110) of clinics. SLTs reported that 47.3% (52/110) of radiographers have received specialist training in VFS, 18.2% have no specialist training and 25% of respondents did not know. The number of radiographers with specialist training is significantly associated with the presence of a radiologist [*Χ*^2^(16) = 28.7, *p* = 0.02]. In radiologist led clinics, 30.6% (11/36) of radiographers have received specialist training, whereas in a practitioner-led clinic 65.8% (48/73) of radiographers have received specialist training.

#### Ionising Radiation Medical Exposure Regulation (IRMER) Operator

IRMER Operators are legal duty holders who are entitled to carry out practical aspects of a medical exposure. Practical aspects include the physical conduct of the exposure and other supporting aspects that have an influence on radiation dose to the patient. Of the 78 clinics where a radiologist is ‘not’ or ‘only sometimes’ present, the radiographer acts as Operator 65.4% (51/78) of the time, SLT 19.2% (15/78), both 1.3% (1/78), while the remainder of responses were unclear (1.3%), not applicable (7.7%) or not known (5.2%).

#### Operator Training

Of the SLTs that reported that the SLT acts as an Operator, 81.2% (13/16) reported they have received Operator training.

### Assessment Methods

#### Protocols

Standard assessment protocols are used in 47.5% (48/101) of VFS clinics. An in-house protocol is used by 68.9% (33/48) of clinics, Modified Barium Swallow Impairment Profile (MBSImP) is used by 20.8% (10/45), ‘Logemann’s protocol’ by 2.1% (1/48) and Dysphagia/Aspiration of at Risk Structures Trial protocol (DARS) by 2.1% (1/48).

#### Test Material

A range of textures were reported to be tested during the assessment, including normal diet and fluids. International Dysphagia Descriptors Standardisation Initiative (IDDSI) fluid levels are used more frequently than the UK National descriptors. Several comments were received stating that they were preparing for a change over to IDDSI.

Set recipes are used in 53.4% (54/101) of services for preparing the oral trials, 45.6% (46/101) of services reported they do not use recipes; however, 11% (5/46) of these reported they are working on introducing recipes.

#### Contrast

Both barium and water soluble contrasts are used in VFS. Thirty out of 101 (29.7%) clinics use barium contrast only, 11.9% (12/101) use a water soluble contrast only and 44.6% (45/101) use both. Of importance, 13.9% (14/101) do not know what contrast they used. A breakdown of the different types of contrast used is shown in Supplementary Online Material 2.

62.2% (46/74) of those using barium as a contrast, reported they did not know what percent weight to volume (w/v) or volume to volume (v/v) contrast to fluid they use. 18.9% (14/74) gave an unclear or variable response. Of those that did respond 6 use 40% w/v, 4 use less than 40% w/v and 4 use more than 40% w/v.

### VFS Analysis Methods

#### Staff

Two SLTs analyse the VFs in 58.2% (64/110) of clinics, one SLT analyses in 30.0% (33/110) of clinics, one or two SLTs analyse in 9.1% (10/110) of clinics and three SLTs analyse in 0.9% (1/110) of clinics.

Radiographers assist in the interpretation in 45.5% (50/110) of the clinics; of these 50, 36% (18/50) assist in the analysis of the oesophageal stage, 4% (2/50) of the oropharyngeal stage and 60% (30/50) in both stages. Radiographers are less involved in interpretation when a Radiologist is present in clinic [*Χ*^2^(4) = 16.4, *p* = 0.003].

#### Protocols

**Fig. 2:**
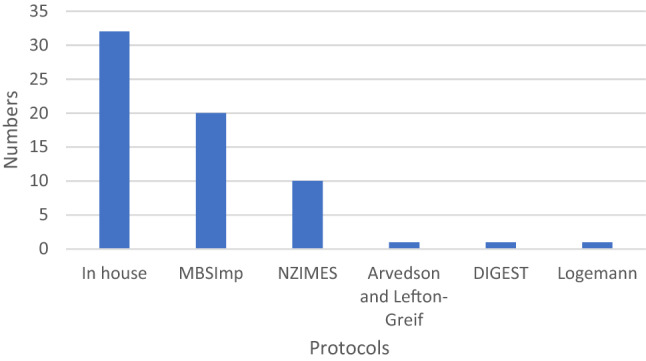
Protocols used in analysis of videofluoroscopy. *NZIMES* New Zealand Index for Multidisciplinary Evaluation of Swallowing, *DIGEST* Dynamic Imaging Grade of Swallowing Toxicity, *MBSImP* Modified Barium Swallow Impairment Profile

A standard protocol for analysis is used in 56.4% (57/101) of clinics, 75.4% (43/57) of those that do, report it is used consistently across VFS studies. Most of the respondents use in-house analysis protocols. Figure 2 shows the range of protocols used.

Frame-by-frame analysis is used in analysis in 58.4% (59/101) of clinics, a further 6.9% (7/101) reported using it sometimes. Of those that said they do not use it, 60.6% (20/33) said they are unable to whereas 33.3% (11/33) said they do not need to.

#### Rating Scales

**Fig. 3:**
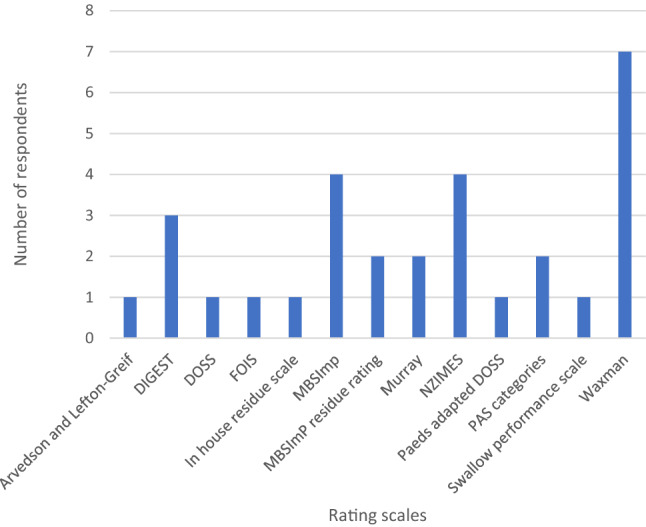
Rating scales used in addition to penetration aspiration scale in VFS analysis. *DIGEST* Dynamic Imaging Grade of Swallowing Toxicity, *DOSS* Dysphagia Outcome and Severity Scale, *FOIS* Functional Oral Intake Scale, *MBSImP* Modified Barium Swallow Impairment Profile, *NZIMES* = New Zealand Index for Multidisciplinary Evaluation of Swallowing, *PAS* Penetration Aspiration scale

88.1% (89/101) of respondents reported they use at least 1 rating scale, 28.7% (29/101) reported using more than one scale, 9.9% (10/101) did not respond and 1% (1/101) reported they do not use rating scales. 87% reported using the Penetration Aspiration Scale (PAS). Figure 3 shows the range of other scales used. Two respondents reported using the Murray rating scale, a reference for which could not be located.

### Technical Configuration

#### Imaging Mode

50.5% (51/101) reported using continuous screening during VFS, 21.8% (22/101) use pulsed screening, 1% (1/101) use both and 26.7% (27/101) did not know.

The most common pulse rate of those that use pulsed imaging is 15 pps (47.8% 11/23), followed by 13% (3/23) using a mix of 30 and 15 pps, 4.3% (1/23) use less than 15 pps and the same amount use 30 pps. The remaining 30.4% (7/23) did not know or did not respond. Several contradictory responses were received.

#### Frame Rate of Acquisition

29.7% replied that they were unaware of the frame rate they use. The most frequently reported frame rate is 15 fps (30.7%), followed by 30 fps (21.8%). Supplementary Online Material 3 shows the imaging mode and frame rate reported by respondents.

#### Recording System

Most respondents reported using a hospital system to record the VFS data (58.4%), followed by DVD (25.7%). The remainder use other digital systems and multiple storage. Of those that reported using a hospital system solely or with another recording system 34.3% (23/67) use a frame rate of 15 fps, 22.4% (15/67) use 30 fps.

Again, there were inconsistencies in the responses, for example eight respondents reported using DVD to record data at 30 fps—which is not possible in the UK.

#### Screening Time

46.5% (47/101) did not know their usual average screening times or did not respond to the question. Of those that responded, screening times ranged from 0.7 min up to 10 min. 46% (25/54) screen for 1–3 min and 44.4% screen for 3–5 min. See Table 1 for details of screening times in time brackets. Median screening time was 3 min (IQR 2.5–3.5).

**Table 1:** Usual VFS screening times grouped into time brackets

Screening times	Number (%)
< 1 min	1
1.1–3 min	25
3.1–5 min	24
5.1–8 min	3
8.1–10 min	1
N/K	35
No response	12
Total	101

35.6 (36/101) reported they have a set maximum screening time and 35.6% (36/101) reported they did not, the remainder reported they did not know (26.7%), their response was unclear (1.0%) or they did not respond (1%). Max screening times ranged from 2 to 7 min and the majority 38.2% (13/34) reported this is set at 5 min. 29.4% (10/34) did not know what it was. Mean maximum screening time was 5 min (IQR 4–5).

#### Data Quality

**Fig. 4:**
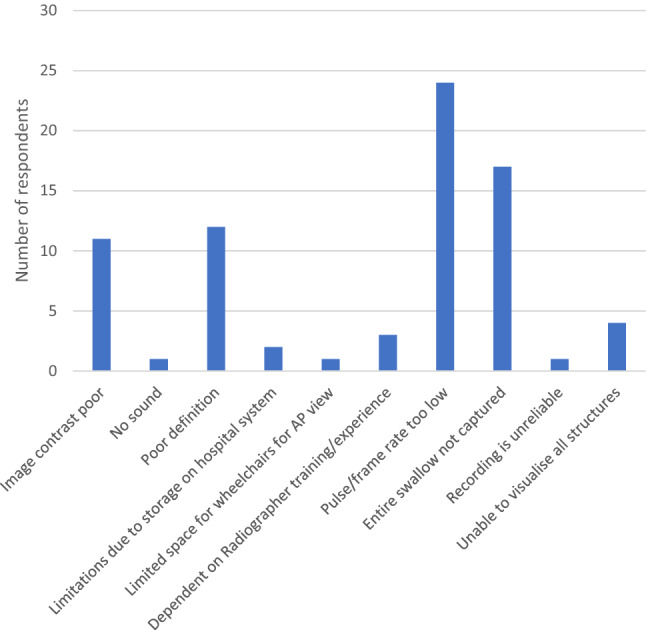
Problems encountered with videofluoroscopy analysis

Good quality images were reported by 54.4% (55/101) of respondents with no problems with video analysis. One or more problems with quality were reported by 45.5% (46/101). Figure 4 gives a breakdown of the problems encountered in analysing VFS. The main problems encountered are frame/pulse rate being too low (52.2% 24/46), entire swallow not captured (37.0% 17/46), poor definition (26.1% 12/46) and poor image contrast (24.0% 11/46).

There were no associations regarding who leads the clinic or banding of SLTs and data quality; however, there was a significant association between Radiographers who had received specialist training and data quality [*Χ*^2^(2) = 6.9 *p* < 0.03], 35% more data quality issues were reported with those that had not received training. This appeared to be associated with specific issues of poor definition [*Χ*^2^(2) = 7.0 *p* < 0.03] and the whole swallow not being captured [*Χ*^2^(2) = 8.8 *p* < 0.013].

### Extended Service

50.5% (51/101) reported scanning the oesophagus during the VFS, 26.7% (27/101) scan the oesophageal phase only when there are indications, 5.9% (6/101) scan only if a Radiologist is present and 2.0% (2/101) scan inconsistently. It was more likely for the assessment to include scanning of the oesophagus in a practitioner-led clinic [*Χ*^2^(12) = 41.9 *p* < 0.001]. Lastly, three clinics reported being able to carry out manofluoroscopy (VFS and manometry).

## Discussion

A UK national survey of SLTs involved with VFS clinics was completed to find out how clinics are presently conducted. Since the publication of a UK survey in 2006, RCSLT have updated their VFS guidelines and further research has been conducted, but little is known about how these guidelines and evidence have been embedded into clinical practice.

We found that the majority of clinics are practitioner-led, usually run by two SLTs and a radiographer. This is positive as practitioner-led clinics have been shown to increase access to clinics without compromising safety [9] and reduce clinic costs [10] when compared to Radiologist led clinics.

Around half of clinics use set protocols for assessment, the majority of these being developed in house. The RCSLT position paper on VFS advises the use of a systematic and structured framework for assessment but recognises that it needs to be flexible due to variations in patients in clinic [2]. A handful of published protocols exist and it is unclear from the survey why they are not routinely used in clinics. A similar lack of structured protocols was found in the survey conducted by Power et al. [11].

Recipes are only used in about half of clinics suggesting that in many clinics there may be variability in texture of the oral trials and contrast concentration. It is possible to achieve correct viscosities if systematic mixing protocols are used and are matched to measures of viscosity [7]. Recipes are also needed to standardise the concentration of contrast used to ensure adequate visibility on images without them leaving a coating in the oral or pharyngeal cavities [6]. Consequently the RCSLT VFS position paper suggests caution with contrasts, without specific detail or references [2]. Similar to the Power et al. [11] study, very few respondents knew what concentration they used and several reported using greater than 40% weight to volume of barium sulphate (Ba). This has been found to leave a coating [6] and may be interpreted as residue as a consequence of pharyngeal stage impairment.

Ba and water soluble contrasts are used widely in the clinics surveyed as was also found in the 2006 survey [11]. Water soluble contrasts are also designed to coat so that structures are visible but no studies have been conducted looking at the effects of their concentration on the interpretation of VFS. The RCSLT guidance suggests water soluble contrasts should be considered in patients who are at high risk of aspiration [2] due to concerns that aspiration of barium can result in pulmonary injury as seen in animal studies [12, 13]. However, a separate study found that water soluble contrasts also caused pulmonary injury in rats [14]. This guidance has filtered into clinical practice but questions remain about the use and safety of contrasts suggesting further research is indicated.

The use of rating scales and standardised protocols helps to improve reliability of analysis [15–17]. Rating scales were used frequently; mostly the PAS [18] as recommended by the RCSLT position paper. Just over a quarter used an additional scale which were mostly variations of dysphagia severity, aspiration or residue rating scales. A recognised impairment profile was used by 32 respondents; either the New Zealand Index of Multidisciplinary Evaluation of Swallowing (NZIMES) [19], MBSImP [16] or the Arvedson and Lefton-Greif [20] for paediatric clinics. Analyses and reports describing swallowing impairments improve the reliability of VFS interpretation and ongoing SLT management [21]. Frame-by-frame analysis is recommended to improve reliability of interpretation [2, 16], but just over 40% of respondents reported they did not use it, with several reporting they did not deem it necessary. The survey did not include questions around how reports are written and recommendations are made to patients, this would be useful to include in future studies.

Continuous imaging was the most commonly reported mode of imaging. Continuous imaging can be captured and recorded onto a digital device at up to 30 fps or onto DVD at 25 fps. Most of those that reported continuous imaging reported capturing 15 fps. The most commonly reported pulse rate for those reporting using pulsed fluoroscopy is 15 pps. Analysing images at a lower pulse or frame rate changes the temporal resolution of the VFS [5] and reducing from 30 to 15 fps can result in less accurate interpretation [3, 4]. This is not surprising given that a swallow occurs in less than one second [22]. Most respondents use a hospital imaging system such as PACS to store data, which often has a size limitations and may be one explanation for lower frame rates. Another explanation is concern regarding radiation dose. However, this may be overstated. Bonilha and colleages showed that a clinical VFS set to continuous screening at 30 fps using the MBSImP (13 bolus protocol and trial of strategies which takes an average of 2.9 mins to administer) results in an average effective dose of 0.27 mSv [23]. Effective doses between 0.1–1 mSv are regarded as low dose [24], equal to 6–7 weeks of background radiation based on the UK average [25].

Screening time is another factor that influences radiation dose and should not be excessively long. However, the assessment needs to be of sufficient duration to ensure the impairment is described, the risks identified and management strategies trialled. The average screening time reported in the survey was 3 min and it ranged from 0.7 up to 10 min. Only 4 respondents reported average screening times greater than 5 mins. These data are unlikely to be accurate as it is based on the respondent’s best guesses. However, it provides an insight into the clinical settings and is akin to other studies reporting average screening times [3, 26, 27] and UK national diagnostic reference levels indicating the upper boundaries for screening time at 3.5 min [28].

A high percentage of respondents reported not knowing information regarding their assessment procedures, such as type of contrast, contrast concentration, and the operation of the fluoroscopy equipment such as, acquisition mode, pulse rate, frame rate and screening times. Given that these factors affect the quality of the assessment and analysis and may result in inaccurate interpretation, SLTs should be familiar with them. Many SLTs are trained in-house and even if SLTs attend external VFS courses the focus may be on assessment and interpretation rather than technical clinic details [1]. Likewise, Radiologists and Radiographers may not have specialist knowledge of oropharyngeal dysphagia or be aware of the implications of the differing recommended technical configurations of VFS as the need for such a high temporal resolution is unique to VFS. Our data highlight that little has changed since the Power et al. survey, that specialist interdisciplinary training for VFS practitioners continues to be important, especially given that both SLTs and Radiographers are involved in assessment and analysis processes. This also corresponds with previous work by Nightingale et al., who suggested that for VFS clinics to be conducted according to the evidence base, specialist training of SLTs and Radiographers is required [29]. Additionally, collaboration with radiology and radiography to develop clear national, and local, VFS guidance, may help to address some of these concerns. A similar conclusion was reached following the survey carried out by Power et al. [11].

Patients who present with oropharyngeal dysphagia, may alternatively or additionally have an oesophageal stage impairment [30]. Oesophageal screening allows for a more thorough assessment and timely referral for further investigation when abnormalities are found [31]. Only in recent years has oesophageal screening been discussed in the literature, as part of, or an adjunct to, VFS assessment [16, 32]. Our study found that around half of UK clinics reported screening the oesophagus during VFS, but it is unclear whether clinics are using standardised, validated screening tools which would be important for increasing accuracy and reliability of interpretation.

### Limitations

The number of VFS clinics across the UK is unknown therefore it is impossible to estimate response rate which is important in evaluating the quality of the results and in identifying non-response bias [33]. There was however representation across the UK, therefore good geographical representation has been achieved. A second limitation of web-based surveys is that there may be multiple responses from the same individual or clinic by requesting hospital names it helped to identify these cases. A further limitation of using surveys is the uncertainty of whether the responses received reflect true clinical practices or the knowledge of the respondent. Information about the respondents was not gathered, therefore SLT experience is not known, a factor which could impact on knowledge based questions. Finally, several respondents had difficulties accessing the survey as some NHS trusts do not allow access to web-based surveys.

## Conclusion

To achieve accurate, reliable and repeatable results from VFS investigations, certain operational criteria need to be met. These criteria are described in National VFS guidelines and in research literature. Our research shows that UK VFS clinics have implemented some, but not all, of these criteria. Data quality was often reported as an issue, suggesting that technical processes are not optimal. Storage limitations for VFS recordings may contribute to reduced quality of captured images. SLTs demonstrated reduced awareness of the technical configuration of their VFS clinic. Specialist training for radiologists, radiographers and SLTs may help to address this. Barriers would need to be explored further, but collaboration with radiology at a national level and the creation of more detailed and up-to-date national guidelines may help to ensure current knowledge about best practice filters into clinical practice. Further research comparing water soluble contrasts with barium in VFS analysis is required as little is known about these products, which are widely used in the UK. This survey has explored practices in the UK; however, further research into international VFS clinic practice would be beneficial.

## Electronic supplementary material

Below is the link to the electronic supplementary material.

Supplementary file1 (PDF 189 kb)

Supplementary file2 (PDF 101 kb)

Supplementary file3 (PDF 79 kb)
