# Supplementary material for: The Landscape of Videofluoroscopy in the UK: A Web-Based Survey
Source: Dysphagia. 2020 May 16;36(2):250–8. doi: 10.1007/s00455-020-10130-1 (PMC8004508; doi:10.1007/s00455-020-10130-1)
Supplement: Supplementary file 1 — Supplementary file1 (PDF 189 kb) [file 455_2020_10130_MOESM1_ESM.pdf]

Videofluoroscopy (VFS) Clinic Survey

*Required

1. **What is your profession?** *
Mark only one oval.
   - Speech and Language Therapist
   - Radiologist
   - Radiographer

2. **Hospital name** *

____

3. **City** *

____

Who is involved in VFS?

If you have more than 1 type of VFS clinic - please answer the questions for your main clinic. You will have a chance to give answers for any further clinics your service runs in the next section.

4. **How many VFS slots are available per week?**

____

5. **How many Speech and Language Therapists (SLTs) conduct the clinic?**
Mark only one oval.
   - 1
   - 2
   - Other: _______________

6. **What Band SLT(s)?**

____

7. **How many SLTs are involved with analysis?**
Mark only one oval.
   - 1
   - 2
   - Other: _______________

8. **Is a Radiologist present?**
Mark only one oval.
   - Yes
   - No
   - Other: _______________

9. **If not is a Radiologist available to review images?**
Mark only one oval.

- Yes
- No
- Other: ____________

10. **Is a Radiographer present?**
Mark only one oval.

- Yes
- No
- Other: ____________

11. **Has the Radiographer received specialist VFS training?**
Mark only one oval.

- Yes
- No
- Don't know
- N/A
- Other: ____________

12. **How much is the Radiographer involved in interpreting?**
Tick all that apply.

- [ ] Not at all
- [ ] Assist in the analysis of the oesophageal stage
- [ ] Assist in the analysis of the oropharyngeal stages
- [ ] Both oropharyngeal & oesophageal stages
- [ ] Other: ____________

13. **If the VFS is SLT led, who acts as the IRMER Operator?**
Mark only one oval.

- SLT
- Radiographer
- Don't know
- N/A
- Other: ____________

14. **Do you have another clinic set up in a different way?**
Mark only one oval.

- Yes
- No *Skip to question 24.*

Second clinic operated differently?

This time answer the questions based on your second clinic, if you do not have a second clinic ignore this section.

15. **How many Speech and Language Therapists (SLTs) conduct the clinic?**
Mark only one oval.

- 1
- 2
- Other: ____________________

16. **What Band SLT(s)?**

____

17. **How many SLTs are involved with analysis**
Mark only one oval.

- 1
- 2
- Other: ____________________

18. **Is a Radiologist present?**
Mark only one oval.

- Yes
- No
- Other: ____________________

19. **If not, is a Radiologist available to review images?**
Mark only one oval.

- Yes
- No
- N/A
- Other: ____________________

20. **Is a Radiographer present?**
Mark only one oval.

- Yes
- No
- Other: ____________________

21. **Has the Radiographer received specialist VFS training?**
Mark only one oval.

- Yes
- No
- Don't know
- Other: ____________________

22. **How much is the Radiographer involved in interpreting?**
Tick all that apply.

- [ ] Not at all
- [ ] Assist in the analysis of the oesophageal stage
- [ ] Assist in the analysis of the oro-pharyngeal stage
- [ ] Both oropharyngeal & oesophageal stages
- [ ] Other: ______________

23. **If the VFS is SLT led, who acts as the IRMER operator?**
Mark only one oval.

- SLT
- Radiographer
- Don’t know
- N/A
- Other: ______________

Standard Protocols

24. **Do you follow a standardized protocol for the assessment?**
Mark only one oval.

- Yes
- No
- Don't know
- Other: ______________

25. **Protocol details**
Please write the name of any published protocols you use or 'in house' if developed in house.

____

26. **Is it used consistently by all therapists?**
Mark only one oval.

- Yes
- No
- N/A
- Other: ______________

27. **Do you use a standardized protocol for interpreting/ reporting?**
Mark only one oval.

- Yes
- No
- Don't know

28. **Analysis protocol**
Please write the name of any published protocols you use or 'in house' if developed in house.

29. **Is it used consistently by all therapists?**
Mark only one oval.

- Yes
- No
- N/A
- Other:

30. **Which rating scales do you use?**
E.G for aspiration, residue or impairment

31. **Do you do frame by frame analysis?**
Mark only one oval.

- Yes
- No, don't need to
- No, unable to
- Other:

32. **What % contrast to fluid/food do you use ?**
I.e. what weight/volume to volume do you use? If you do not know, write N/K below

33. **What textures do you trial?**

Tick all that apply.

- [ ] Normal/thin fluids
- [ ] UK descriptors Stage 1/Syrup
- [ ] UK descriptors Stage 2/Custard
- [ ] UK descriptors Stage 3/Pudding
- [ ] IDDSI Level 1 fluids
- [ ] IDDSI Level 2 fluids
- [ ] IDDSI Level 3 fluids
- [ ] Puree diet
- [ ] Premashed/Mince and moist diet
- [ ] Forkmashable/Soft and bitesized diet
- [ ] Normal/Regular diet
- [ ] Other: ______________________

34. **What contrast do you use?**

Please give details of all the contrasts you use. If unknown please write N/K below.

____

____

____

____

____

35. **Do you have set recipes?**

Do you have a standard set of detailed recipes for each texture trialled i.e. 60ml of juice, 40ml barium, 1/2 scoop thickener?

Tick all that apply.

- [ ] Yes
- [ ] No
- [ ] Other: ______________________

Xray set up

36. **Do you use fluoroscopy only?**

Mark only one oval.

- ( ) Yes
- ( ) No
- ( ) Don't know
- ( ) Other: ______________________

37. **Do you use fluorograb?**
Mark only one oval.

- Yes
- No
- Don't know
- Other: ____________________

38. **Do you use spot images?**
Mark only one oval.

- Yes
- No
- Don’t know
- Other: ____________________

39. **Do you scan the oesophagus?**
Mark only one oval.

- Yes
- No
- Other: ____________________

40. **Do you use continuous or pulsed fluoroscopy?**
Mark only one oval.

- Continuous
- Pulsed
- Don’t know
- Other: ____________________

41. **What fluoroscopy pulse rate do you typically use?**
Mark only one oval.

- Less than 15pps
- 15pps
- 30 pps
- Don't know
- N/A
- Other: ____________________

42. **What frame rate do you use?**
Mark only one oval.

- Less than 15fps
- 15fps
- 25fps
- 30fps
- Don't know
- Other: ____________

43. **How do you record data?**
Mark only one oval.

- Hospital system
- Kay Pentax
- Video
- DVD
- Digital
- Other: ____________

44. **Do you record sound?**
Mark only one oval.

- Yes
- No
- Unable to
- Don't know
- Other: ____________

45. **What fluoroscopy mode do you typically use?**
Mark only one oval.

- Low
- Normal
- High
- Don’t know
- Other: ____________

46. **What is the age of the fluoroscopy X-ray system?**
Mark only one oval.

- < 5 years
- 5 to 10 years
- Older than 10 years
- Don’t know
- Other: ____________

47. **Do you use specialised fluoroscopy protocols for paediatric patients (if relevant)?**
Mark only one oval.

- No paediatric clinic
- Yes
- No
- Don’t know
- Other: ____________

48. **If yes please specify**

____
____
____
____
____

49. **Have you received any IRMER training as an Operator?**
Mark only one oval.

- Yes
- No
- Don’t know
- Other: ____________

50. **What is your usual screening time?**
If you do not know, write N/K below. If you know an average range i.e. 2-4 minutes please write this

____

51. **Do you have a maximum screening time?**
Mark only one oval.

- Yes
- No
- Don't know
- Other: ____________

52. **If yes, what is it?**

____

53. **Do you have any problems with image quality for VFS analysis?**
Tick all that apply.

- [ ] No problems/Good quality image
- [ ] Unable to visualise all structures
- [ ] Pulse/frame rate too low
- [ ] Image contrast poor
- [ ] Recording does not capture swallow from start to end
- [ ] Poor definition
- [ ] Other: ______________________

54. **Is your department able to carry out manofluoroscopy?**
Manofluoroscopy = manometry + videofluoroscopy
Mark only one oval.

- ( ) Yes
- ( ) No
- ( ) Don't know
- ( ) Other: ______________________

Other comments

Please write any other comments related to your videofluoroscopy clinic which you wish to share.

55. **Comments**

____
____
____
____
____

Powered by
Google Forms
