# Supplementary material for: The Landscape of Videofluoroscopy in the UK: A Web-Based Survey
Source: Dysphagia. 2020 May 16;36(2):250–8. doi: 10.1007/s00455-020-10130-1 (PMC8004508; doi:10.1007/s00455-020-10130-1)
Supplement: Supplementary file 2 — Supplementary file2 (PDF 101 kb) [file 455_2020_10130_MOESM2_ESM.pdf]

The Landscape of Videofluoroscopy in the UK: A web-based survey
Dysphagia
Benfield, J.K., Michou, E., Everton, L.F., Mills, C., Hamdy, S., Bath, P.M., England, T.J.

Electronic Supplementary Material 2

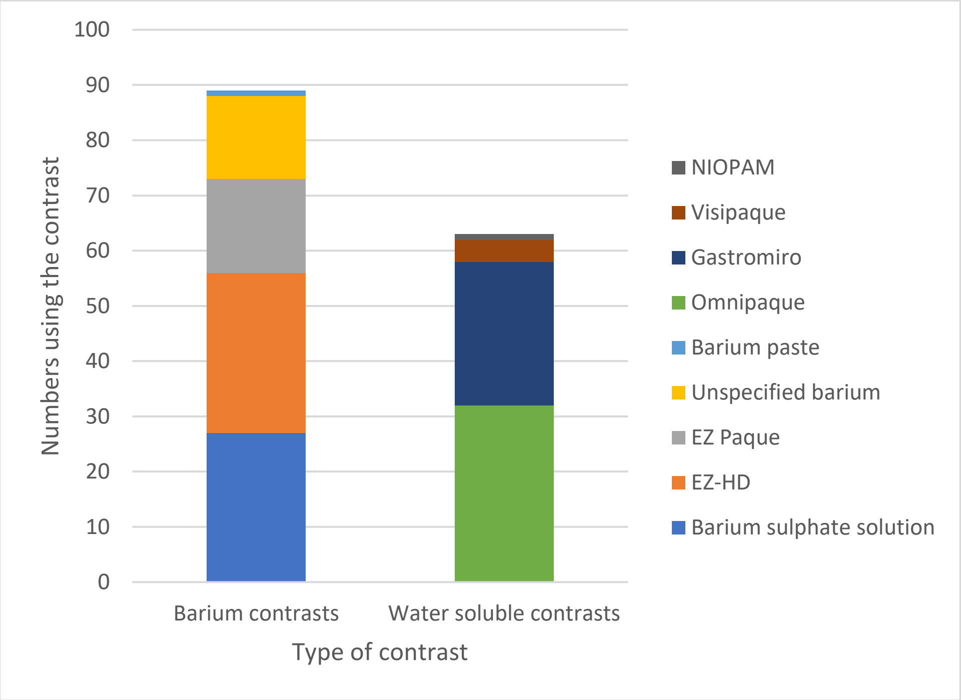

The types and varieties of contrast and their frequency of use in VFS clinics.
