# Supplementary material for: The Landscape of Videofluoroscopy in the UK: A Web-Based Survey
Source: Dysphagia. 2020 May 16;36(2):250–8. doi: 10.1007/s00455-020-10130-1 (PMC8004508; doi:10.1007/s00455-020-10130-1)
Supplement: Supplementary file 3 — Supplementary file3 (PDF 79 kb) [file 455_2020_10130_MOESM3_ESM.pdf]

The Landscape of Videofluoroscopy in the UK: A web-based survey
Dysphagia

Benfield, J.K., Michou, E., Everton, L.F., Mills, C., Hamdy, S., Bath, P.M., England, T.J.

Electronic Supplementary Material 3: Imaging mode and frame rate

	**Frame rate (frames per second)**							
**Type of screening**	**Less than 15**	**15**	**25**	**30**	**Mix of 30 and ≤15**	**Don't know**	**No response**	**Total**
Continuous	5	14	2	14	2	13	1	51
Pulsed	2	10		3	1	6		22
Both		1						1
Don't know	5	6		5		11		27
**Grand Total**	**12 (11.9%)**	**31 (30.7%)**	**2 (2%)**	**22 (21.8%)**	**3 (3%)**	**30 (29.7%)**	**1 (1%)**	**101**
